# Sanitation in urban areas may limit the spread of antimicrobial resistance via flies

**DOI:** 10.1371/journal.pone.0298578

**Published:** 2024-03-20

**Authors:** Drew Capone, Oliver Cumming, Abeoseh Flemister, Victor Ilevbare, Seth R. Irish, Ishi Keenum, Jackie Knee, Rassul Nala, Joe Brown

**Affiliations:** 1 Department of Environmental and Occupational Health, Indiana University, Bloomington, Indiana, United States of America; 2 Department of Disease Control, London School of Hygiene and Tropical Medicine, London, United Kingdom; 3 Roy Blunt NextGen Precision Health, University of Missouri, Columbia, Missouri, United States of America; 4 Department of Radiology, University of Missouri, Columbia, MO, United States of America; 5 Department of Biology, University of North Carolina at Chapel Hill, Chapel Hill, North Carolina, United States of America; 6 Epidemiology and Public Health Department, Swiss Tropical and Public Health Institute, Allschwil, Switzerland; 7 Department of Civil, Environmental and Geospatial Engineering, Michigan Technological University, Houghton, Michigan, United States of America; 8 Ministério da Saúde de Moçambique, Instituto Nacional de Saúde, Maputo, Mozambique; 9 Department of Environmental Sciences and Engineering, University of North Carolina at Chapel Hill, Chapel Hill, North Carolina, United States of America; Kerman University of Medical Sciences, ISLAMIC REPUBLIC OF IRAN

## Abstract

Synanthropic filth flies are common where sanitation is poor and fecal wastes are accessible to them. These flies have been proposed as mechanical vectors for the localized transport of fecal microbes including antimicrobial resistant (AMR) organisms and associated antimicrobial resistance genes (ARGs), increasing exposure risks. We evaluated whether an onsite sanitation intervention in Maputo, Mozambique reduced the concentration of enteric bacteria and the frequency of detection of ARGs carried by flies collected in household compounds of low-income neighborhoods. Additionally, we assessed the phenotypic resistance profile of *Enterobacteriaceae* isolates recovered from flies during the pre-intervention phase. After fly enumeration at study compounds, quantitative polymerase chain reaction was used to quantify an enteric 16S rRNA gene (i.e., specific to a cluster of phylotypes corresponding to 5% of the human fecal microflora), 28 ARGs, and Kirby Bauer Disk Diffusion of *Enterobacteriaceae* isolates was utilized to assess resistance to eleven clinically relevant antibiotics. The intervention was associated with a 1.5 log_10_ reduction (95% confidence interval: -0.73, -2.3) in the concentration of the enteric 16S gene and a 31% reduction (adjusted prevalence ratio = 0.69, [0.52, 0.92]) in the mean number of ARGs per fly compared to a control group with poor sanitation. This protective effect was consistent across the six ARG classes that we detected. *Enterobacteriaceae* isolates–only from the pre-intervention phase–were resistant to a mean of 3.4 antibiotics out of the eleven assessed. Improving onsite sanitation infrastructure in low-income informal settlements may help reduce fly-mediated transmission of enteric bacteria and the ARGs carried by them.

## Introduction

Antimicrobial resistance (AMR) is a serious and growing public health threat, with nearly 1.3 million deaths attributable to AMR annually (2019 estimate) [1]. Research on infection prevention and control has historically focused on clinical settings, where outbreaks of resistant organisms have frequently resulted in patient deaths [2] and emerging “superbugs” are increasingly prominent. Animal husbandry has also become an increasing area of focus as large quantities of antibiotics are often used in livestock production. While these areas are critical for infection control, water, sanitation, and hygiene (WASH) has been increasingly recognized as a contributor to AMR evolution and dissemination [3–5]. Utilizing a One Health lens–that considers the overlap between human health, animal health and the environment–enables a more systems level approach to combat the spread of AMR. Understanding the role of antimicrobial resistant bacteria (ARB) transmitted by insects–as a direct pathway from human waste to consumption–is critical to potentially mitigating its spread.

Filth flies–including house flies (*Musca domestica*) and green bottle flies (e.g., *Lucilia sericata)*–feed on and can lay their eggs in fecal wastes [6–8]. During feeding, ARB may attach to a fly’s integument or be ingested into the alimentary canal. The rapid proliferation of flies–a single female house fly may lay up to five batches of 100–150 eggs during its one-to-three-month lifespan [8]–and their frequent feeding produces a high potential for the dissemination of resistant organisms when they land on food and household surfaces [9]. House flies and bottle flies frequently vomit and may re-ingest food, because this process helps with digestion. Where food is plentiful, house flies may defecate as often as every four and half minutes [8]. Flies are also fastidious at preening their wings, legs and abdomen, which may dislodge organisms onto food or surfaces.

Improving sanitation may reduce the environmentally mediated transmission of enteric pathogens and other fecal microbes via well-defined pathways [10], including flies. First, some sanitation infrastructure–such as ventilated improved pit latrines and pour flush systems–provides a physical barrier (e.g., a water seal or a mesh cover over a ventilation pipe) that may reduce fly breeding and the potential for flies to transport antimicrobial resistant bacteria (ARB) and their ARGs to the environment. Fewer flies in the living environment may then reduce the risk of enteric infection among those living nearby [11]. Subsequently limiting the need for antibiotics which exhibit pressure on microbial communities to acquire or evolve resistance genes. However, periodic maintenance or replacement of the physical barriers present in onsite sanitation systems is necessary to ensure proper performance.

Filth flies are highly prevalent in low-income, informal communities where sanitation infrastructure is lacking [12, 13]. Studies conducted in Kenya [14], India [15], Bangladesh [16], and in the United States [17] observed one or more flies at 50–100% of dwellings. Yet, limited evidence exists on whether and to what extent flies play a role in the transport of fecal bacteria in such settings via ARB and ARGs. Further, the potential for improved sanitation infrastructure to reduce ARB and ARGs carried by flies is unclear [18]. Elucidating the relationship between improved onsite urban sanitation improvements and ARB carried by flies may provide important evidence to support more effective programs to limit the environmental spread of AMR via enhanced vector control.

The Maputo Sanitation (MapSan) Trial was a controlled before-and-after trial that evaluated the effects of an urban onsite sanitation intervention on child health and environmental fecal contamination in Maputo, Mozambique. The trial’s primary, secondary, and other outcomes have been reported elsewhere [19–28]. The sanitation intervention was not associated with a difference in any health outcome measured in children (i.e., enteric pathogen carriage, 7-day diarrhea recall, height-for-age, weight-for-age) [21]. However, substantial reductions in *Shigella* and *Trichuris* infection among children born after the delivery of the intervention compared to children of similar ages at baseline were observed [21]. Previous MapSan studies also assessed the impact of the intervention on environmental fecal contamination. Modest reductions of enteric pathogens in soils and fecal sludges were observed [29, 30], but the intervention was not associated with reductions in fecal source tracking markers from several environmental matrices [31]. Environmental AMR has not previously been assessed as part of the MapSan Trial.

Our research aims were to: (i) assess the clinically relevant ARG profile carried by flies and the phenotypic profile of *Enterobacteriaceae* spp. isolates recovered from flies; and (ii) to assess the impact of an urban onsite sanitation intervention on enteric bacteria and ARGs detected in flies from low-income neighborhoods in Maputo, Mozambique.

## Methods

### Maputo sanitation trial

We conducted the Maputo Sanitation Trial in 16 low-income, informal neighborhoods in Maputo, Mozambique [21]. In study neighborhoods housing and water, sanitation, and hygiene (WASH) conditions are poor, the burden of enteric disease is high, indiscriminate use of antibiotics is common, and population density is 15,000–25,000 per square kilometer [21, 29]. A non-governmental organization (NGO) delivered the intervention to compounds, which were occupied by two or more households that shared sanitation and a common outdoor living space where daily activities occurred (e.g., cooking, cleaning, and children’s play activities). Intervention systems were built inside the compound boundary and were part of the households’ living environment. The intervention infrastructure contained physical barriers–including mesh netting over ventilation pipes and water-seal toilets–that reduced the potential for flies to breed in the fecal sludge in the septic tank. Control compounds were concurrently enrolled from the same or adjacent neighborhoods as intervention compounds and continued using existing shared sanitation. Detailed descriptions of the inclusion criteria and the sanitation intervention are described elsewhere [21, 32].

### Data collection

Trained field enumerators visited control and intervention compounds at baseline (i.e., pre-intervention, April 2015—March 2016) and 12-months post-intervention (April 2016—March 2017). The enumerators consented caregivers of children enrolled into the overall MapSan trial [21] and administered a survey with the caregiver about household demographics and behaviors. During household visits enumerators collected a convenience sample of flies at latrine entrances and food preparation areas. Field workers used paper traps covered in a sticky material to collect flies at two locations in 50 control and 50 intervention compounds at baseline and 12-months following delivery of the intervention. Enumerators hung rectangular blue sticky traps (pre-intervention: non-baited 5” x 7”, Suterra, Bend, Oregon; post-intervention: non-baited 5.5” x 9.5”, Great Lakes IPM, Vestaburg, Michigan) at least 1.5 meters off the ground and within one meter of the latrine entrance and the food preparation area. The fly trap model was changed for the 12-month follow-up because the manufacturer discontinued the product. After passive fly collection for 24 hours, field workers returned and enumerated the number of flies on each trap. Flies were carefully removed from the trap using tweezers that were sterilized with 10% bleach and 70% ethanol between compounds but not between flies to minimize the time spent at people’s homes. All flies caught on a trap were collected into Whirl-Pak bags (Nasco, Fort Atkinson, Wisconsin) pre-intervention or sterile 15-mL centrifuge tubes (VWR, Radnor, Pennsylvania) 12-months post-intervention. Flies were stored on ice and transported to the Ministry of Health in Maputo, Mozambique. Samples were deposited into a freezer at -80°C on the same day as collection and were shipped from Maputo, Mozambique to Atlanta, Georgia on dry ice (-80°C) with temperature monitoring for later molecular analysis.

### Nucleic acid extraction

We used the Qiagen DNeasy Blood and Tissue Kit (Qiagen, Hilden, Germany) to extract total nucleic acids from 188 individual flies (S1 Text). First, flies were subjected to a pre-treatment step that has been used widely to extract DNA from ticks [33–36]. Then, we proceeded with extraction following the manufacturer’s protocol. For quality control we included two extraction positive controls with each sample and we included at least one negative extraction control on each day of extractions [37].

### TaqMan array card

We assayed bacterial targets and ARGs using a custom TaqMan Array Card (TAC) (ThermoFisher Scientific, Waltham, MA). The TAC tested for an enteric 16S rRNA gene–described in Rousselon *et al*. 2004 [38]–that was designed to detect a cluster of phylotypes, called Fec1, corresponding to 5% of the human fecal microflora [38, 39]. The TAC also included 28 genes conveying resistance to a combination of agriculturally, anthropogenically, and clinically relevant genes which had previously been optimized for the TAC platform by Pholwat *et al*. 2019 (S1 and S2 Tables) [40, 41]. The ARGs corresponded to all eight antimicrobial classes optimized by Pholwat *et al*. 2019 [41]. The card included one gene encoding resistance to aminoglycosides (*armA*), four chloramphenicols (*catA1*, *catB3*, *cmlA*, *floR*), one colistin (*mcr-1*), six fluoroquinolones (*aac6lb_104R*, *aac6lb_104W*, *gyrA83L*, *parC80I*, *qnrA*, *qnrB1*), two macrolides (*ermB*, *mphA*), two tetracyclines (*tetA*, *tetB*), three trimethoprims / sulfamethoxazoles (*dfrA17*, *sul1*, *sul2*), and nine β-lactams (*CTX-M1*, *CTX-M2-M74*, *CTX-M8-M25*, *CTX-M9*, *NDM*, *OXA-1*, *OXA-9*, *SHV*, *VIM*). We performed quantitative PCR (qPCR) using a QuantStudio 7 Flex instrument (Thermofisher Scientific, Waltham, MA). We manually set the threshold by comparing exponential curves and multicomponent plots with the positive control plots (S1 Fig) [29, 42]. Only amplification before a quantification cycle (Cq) of 35 was called as positive for a target.

### Kirby bauer disk diffusion

We performed antimicrobial susceptibility testing according to the Clinical Laboratory Standards Institute (CLSI), with minor adjustments [43]. We randomly selected previously frozen flies collected during the pre-intervention phase of the trial to assess phenotypic resistance of *Enterobacteriaceae* isolates (there were few flies available post-intervention for phenotypic testing because they were nearly all used for molecular analysis). First, we randomly selected two house flies, or two green bottle flies, caught from same compound location, crushed each fly pool in a 15 mL centrifuge tube using a sterile pestle (Kimble Chase, Vineland, NJ), added 12 mL of sterile phosphate buffered saline (PBS, Sigma-Aldrich, St. Louis MO), shook the tube for two minutes, and then allowed the fly solids to settle for approximately ten minutes. Next, we diluted the supernatant in 10-fold increments up to 3 log_10_, transferred 1 mL of the supernatant from each tube in the dilution series to petri dishes containing violet red bile glucose agar (Sigma-Aldrich, St. Louis MO) [44], which is specific to *Enterobacteriaceae*, and then streaked the plates using a flame sterilized inoculating loop. We repeated this plating step using violet red bile glucose agar that contained cefotaxime at 4 μg/mL as a pre-screening step based on the WHO Tricycle Protocol [45]. Following incubation at 37°C for 24 hours, we picked up to three morphologically distinct colonies (S3 Table) per sample from plates with and without cefotaxime, for a total of up to six isolates per sample using flame sterilized inoculating loops. Colonies were speciated by visual inspection (S3 Table), which deviates from CLSI guidelines [43].

We placed individual colonies into glass culture tubes containing 5 mL of sterile tryptic soy broth (TSB, Sigma-Aldrich, St. Louis MO), that did not include antibiotics. Following incubation at 37°C for 24 hours, we combined the bacterial culture with 2 mL of sterile PBS–a deviation from sterile saline used in the CLSI protocol–in a separate glass culture tube to match the turbidity of a 0.5 McFarland turbidity standard. Then we dipped a sterile cotton tipped swab into the glass tube containing PBS-diluted culture and streaked the surface of two petri dishes (90mm diameter, VWR, Radnor, PA) containing Muller Hinton Agar (MHA, Sigma-Aldrich, St. Louis MO). Next, we placed ten antibiotic disks (ciprofloxacin, streptomycin, levofloxacin, chloramphenicol, colistin, azithromycin, tetracycline, trimethoprim-sulfamethoxazole, ampicillin-sulbactam, ceftazidime-avibactam) onto the two plates and flame sterilized the tweezers between each use. Finally, we incubated the MHA plates with the antibiotic disks for 24 hours at 37°C and measured the zones of lysis using calipers to assess if isolates were susceptible, intermediate, or resistant to each antibiotic according to CLSI guidelines (S4 Table) [43, 46].

### Ethics approval

The MapSan Trial protocol was approved by the Mozambican Comité Nacional de Bioética para a Saúde (CNBS), Ministério da Saúde (333/CNBS/14), the Research Ethics Committee of the London School of Hygiene & Tropical Medicine (reference # 8345), and the Institutional Review Board of the Georgia Institute of Technology (protocol # H15160). No additional permits were required.

### Data analysis

The MapSan Trial primary outcome used a difference-in-difference (DID) analysis to assess the impact of the intervention relative to the control [21]. The DID approach is a quasi-experimental approach that typically uses longitudinal data from control and intervention groups. This approach relies on the parallel trend assumption, meaning that the initial difference between the two groups is assumed to remain constant over time. The validity of the parallel trend assumption was previously found valid and reported in the MapSan trial’s primary outcome manuscript [21]. Like the main trial outcome, we used a DID approach to assess the impact of the intervention (i.e., our exposure variable) on our outcomes–that is the prevalence and concentration of the enteric 16S gene, the total number of ARGs detected out of the 28 we assessed and the total number of ARGs in each class of antibiotics–compared to the control group. We used generalized estimating equations (GEE) [47] to fit unadjusted and adjusted regression models with robust standard errors and an exchangeable correlation structure. For count-based outcomes (i.e., total number of ARGs) we used Poisson regression and for continuous outcomes (e.g., concentration of the enteric 16S gene) we used linear regression. We accounted for clustering between compounds because the intervention was implemented at the compound level [48]. *A priori* we decided to adjust regression models for fly mass, fly species, compound location where the fly was caught, compound-level wealth index [49], and compound population. We calculated the compound level wealth index using the Simple Poverty Scorecard for Mozambique, using the average wealth score when multiple households were present [21].

## Results

### Controls

In total, 12 flies from the 188 analyzed using a custom TaqMan Array Card (TAC) were excluded because an extraction control did not amplify as expected. Our eight PCR positive controls–plasmids that contained the primer and probe sequences for each gene target on the TAC [50]–exhibited a positive amplification signal for each target. We did not observe amplification before a C_q_ of 35 for any target in our 12 negative extraction controls.

### Fly prevalence and counts

At baseline–combined from latrine entrances and food preparation areas–we caught a mean of 18 flies per intervention compound (95% confidence interval: 13, 24) and 13 flies per control compound (95% CI: 9.6, 17). At the 12-month follow-up period we caught fewer flies; the mean number of flies caught at intervention compounds was 3.2 (95% CI: 1.8, 4.7) and was 4.5 at control compounds (95% CI: 2.8, 6.2). Disaggregated between compound locations, the intervention reduced mean fly counts at latrine entrances by 69% (adjusted prevalence ratio = 0.31, [0.13, 0.75]) but had no effect on fly counts at food preparation areas (aPR = 0.92, [0.33, 2.6]). Fly counts by month can be found in S5 Table and counts divided phase, arm, and compound location can be found in S6 Table.

### Fecal bacteria

We analyzed 176 flies, of which 90% were houseflies (159/176) and 10% were green bottle flies (17/176). In addition, 66% of flies were caught at food preparation areas (116/176) and 34% at latrine entrances (60/176). The mean concentration of the enteric 16S rRNA gene was 3.5 log_10_ copies per fly (standard deviation: 1.4 log_10_; min: non-detect; max: 7.2 log_10_; Table 1). The onsite sanitation intervention was associated with a 14% reduction in the prevalence of the enteric 16S gene per fly (aPR = 0.86, [0.75, 0.98]) and a 1.5 log_10_ reduction (95% CI: -0.73, -2.3) in the concentration of 16S gene copies per fly.

**Table 1:** Fecal bacteria in flies.

Target	Trial Arm	Baseline	12-month	Unadjusted DID estimate	Adjusted DID estimate
				log_10_ reduction (95% CI)	
enteric 16S gene concentration*	Control	3.5 (1.2)	3.8 (1.3)	**-1.7 (-0.97, -2.5)**	**-1.5 (-0.73, -2.3)**
	Intervention	4.0 (1.1)	2.7 (1.0)		
				PR (95% CI)	aPR 95% (CI)
enteric 16S gene*	Control	98% (55/56)	98% (52/53)	**0.85 (0.73, 0.99)**	**0.86 (0.75, 0.98)**
	Intervention	100% (34/34)	85% (28/33)		

Note: Bold indicates p < 0.05.

*Enteric 16s rRNA gene from Rousselon *et al*. 2004 [38].

### Genotypic resistance

We detected ≥1 ARG from each (176/176) fly we assessed using TAC (Table 2) and detected an ARG from each antimicrobial class in ≥1 flies except for the aminoglycosides (*armA*) and colistin (*mcr-1*). However, these classes only assessed one ARG while other classes assessed multiple ARGs (Table 2). Most detected ARGs were observed at concentrations between 10^4^−10^8^ gene copies per fly (S2 Fig).

**Table 2:** Summary of the detection of antimicrobial resistance genes at baseline and the 12-month follow-up in flies.

Target	Study Arm	Baseline Mean (IQR)	12-Month Mean (IQR)	Unadjusted DID estimate PR (95% CI)	Adjusted DID estimate aPR (95% CI)
β-lactamases (out of 9)	Control	1.5 (2)	1.5 (2)	0.64 (0.35, 1.2)	0.60 (0.33, 1.1)
	Intervention	2.2 (4)	1.4 (2)		
Trimethoprim-sulfonamides (out of 3)	Control	2.5 (1)	2.5 (1)	**0.85 (0.74, 0.98)**	**0.86 (0.74, 0.99)**
	Intervention	2.7 (1)	2.3 (1)		
Tetracycline (out of 2)	Control	1.3 (1)	1.5 (1)	**0.65 (0.45, 0.95)**	0.71 (0.49, 1.0)
	Intervention	1.6 (1)	1.2 (1)		
Macrolides (out of 2)	Control	1.2 (1)	1.3 (1)	**0.54 (0.37, 0.81)**	**0.58 (0.40, 0.86)**
	Intervention	1.6 (1)	0.94 (1)		
Fluoroquinolones (out of 6)	Control	1.4 (1.3)	1.7 (1)	**0.58 (0.34, 0.98)**	0.63 (0.37, 1.1)
	Intervention	1.7 (1)	1.2 (2)		
Chloramphenicol (out of 4)	Control	2.2 (2)	2.3 (1)	**0.65 (0.50, 0.93)**	**0.69 (0.51, 0.95)**
	Intervention	2.8 (2)	2.0 (2)		
Total number of ARGs (out of 28)	Control	10 (7)	11 (7)	**0.67 (0.50, 0.89)**	**0.69 (0.52, 0.92)**
	Intervention	13 (5)	9.0 (5)		

Note: ARGs encoding colistin and aminoglycoside resistance were not detected; DID = difference-in-difference; aPR = adjusted prevalence ratio; CI = confidence interval; IQR = interquartile range; bold indicates p < 0.05

**Fig 1:**
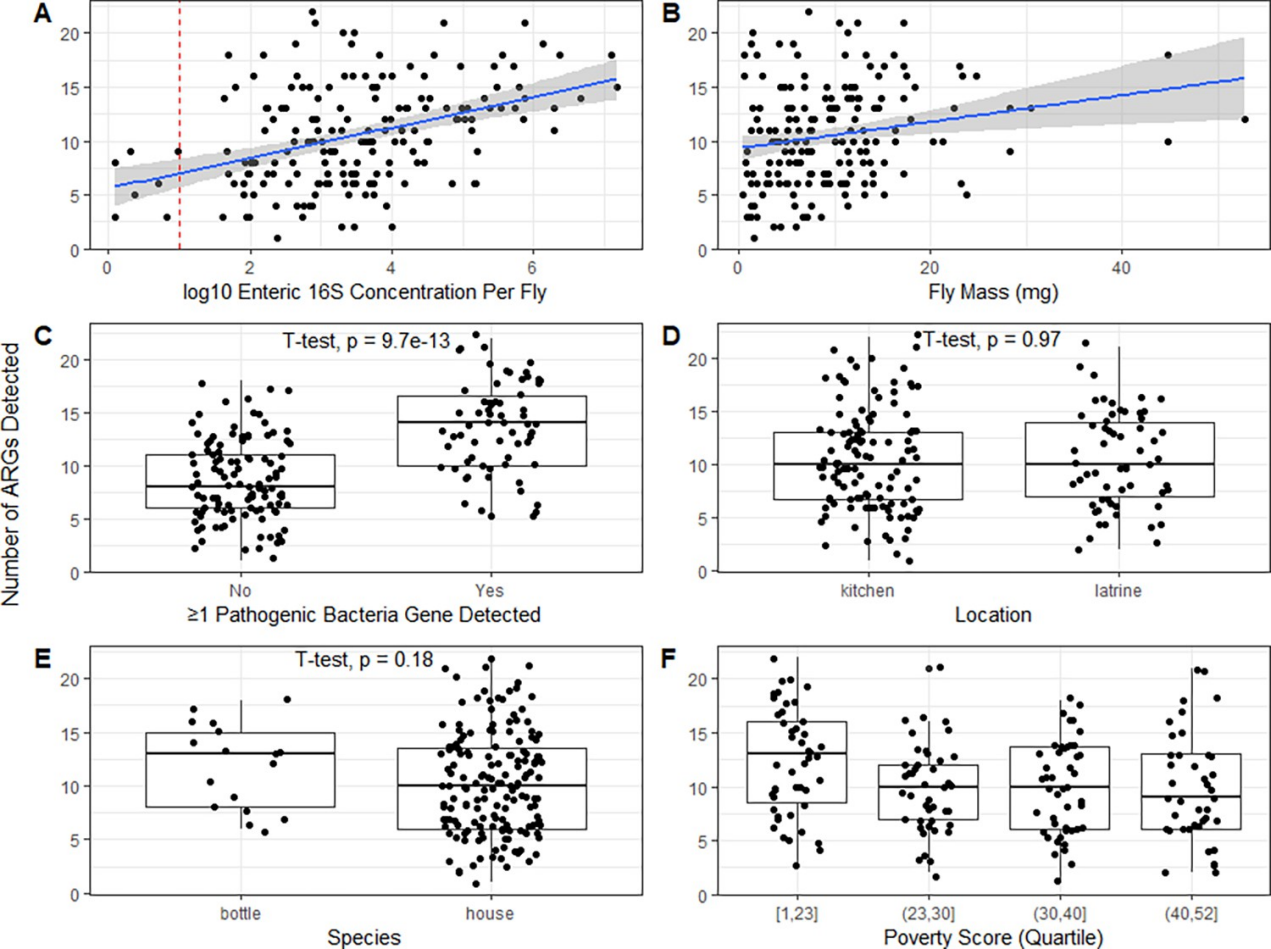
Genotypic resistance carried by flies. Note: In Fig 1A points to the left of the dotted red line are non-detects. Reported p-values from T-tests. Detection of pathogen genes described in Capone *et al*. 2023 [24].

We observed a linear correlation between the concentration of the enteric 16S gene and the number of ARGs detected (Fig 1A), as well as between the fly mass and the number of ARGs detected (Fig 1B). While flies with ≥1 bacterial pathogen gene detected in Capone *et al*. 2023 [24] had a greater number of ARGs detected compared to flies with no bacterial pathogen genes detected (Fig 1C), we observed no difference in the number of ARGs detected in flies caught at different compound locations (Fig 1D) or between fly species (Fig 1E).

### Phenotypic resistance

**Fig 2:**
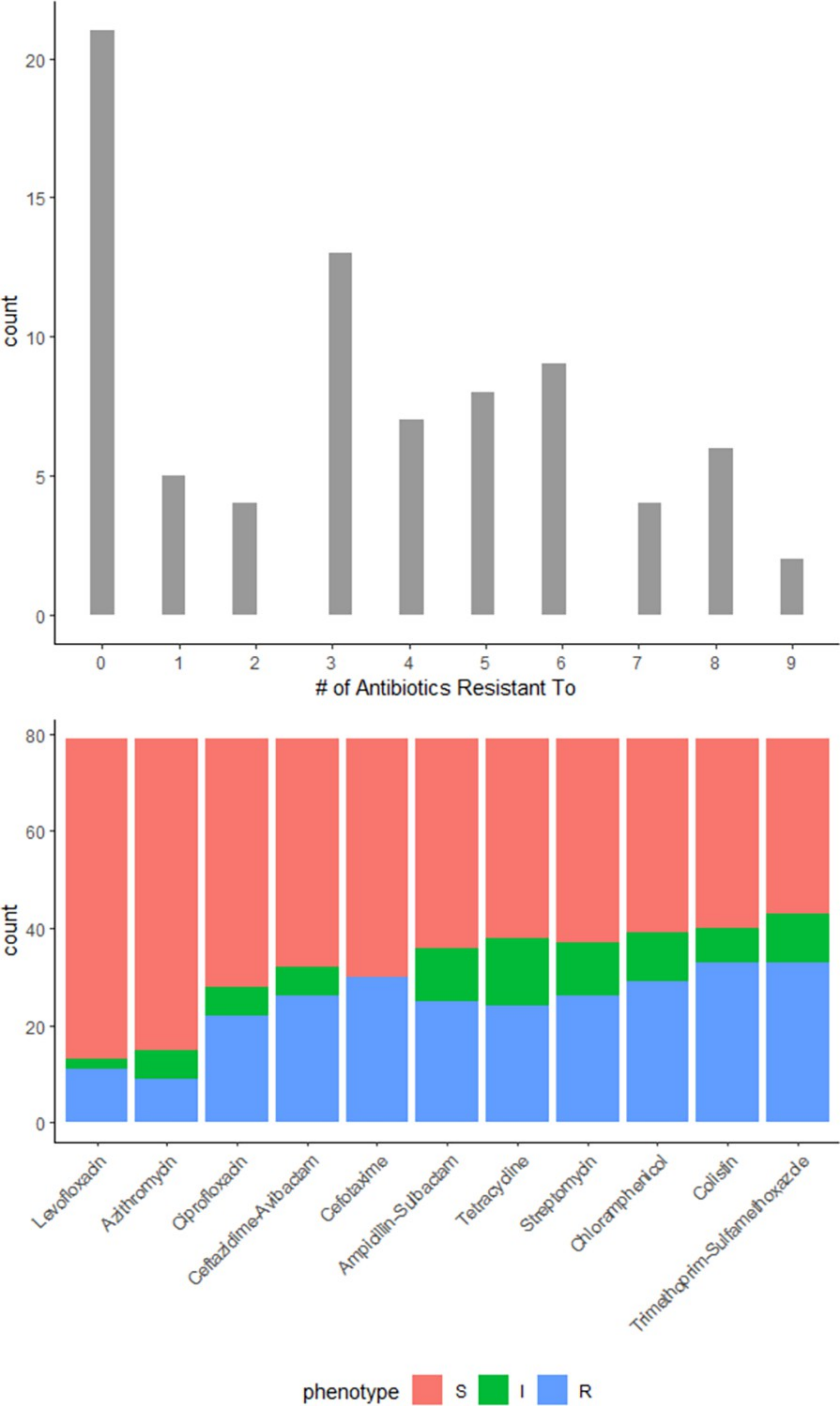
Phenotypic resistance of isolates to eleven antibiotics.

We assessed the phenotypic resistance of 79 *Enterobacteriaceae* isolates from 24 pairs of flies, which were all caught during the pre-intervention phase. We assessed the phenotypic resistance profile of 30 isolates that were resistant to cefotaxime (mean = 1.3 isolates visually identified and screened per pair of flies, range = 0 to 3 isolates per pair of flies) and 49 isolates that were susceptible (mean = 2.0 isolates visually identified and screened per pair of flies, range = 1 to 3 isolates per pair of flies) (S3 Fig). All pairs of flies had at least one morphologically distinct colony on violet red bile glucose agar without cefotaxime, while most pairs of flies (16/24) had at least one morphologically distinct colony on violet red bile glucose agar that contained cefotaxime. The combined 79 isolates were resistant to a mean of 3.4 antibiotics out of the eleven assessed (Fig 2), with 57% (n = 44/79) expressing multi-drug resistance (≥3 antibiotic classes).

Normalizing the results per pair of flies assessed, as some isolates may have been the same species, we found that isolates (mean = 3.3 per pair of flies, range = 1–6 per pair of flies) from each pair of flies were resistant to a mean of 5.6 antibiotics out of the eleven assessed (i.e., the ten assessed via disk diffusion and cefotaxime). Further, 83% (n = 20/24) of the combined isolates per pair of flies expressed multi-drug resistance (≥3 antibiotic classes).

### Impact of the intervention on AMR

From baseline to the 12-month follow-up the mean number of ARGs detected in flies increased from ten to eleven among control compounds but decreased from thirteen to nine among intervention compounds. Using Poisson regression analysis, we found that the intervention was associated with a 31% reduction in the mean number of ARGs per fly (aPR = 0.69, [0.52, 0.92]) compared to controls (Tables 2 and 3). The directionality of this effect remained consistent for the six classes of ARGs that were detected; intervention compounds were associated with a reduction in the mean number of β-lactamases (aPR = 0.60, [0.33, 1.1]), trimethoprim-sulfonamides (aPR = 0.86, [0.74, 0.99]), tetracyclines (aPR = 0.71, [0.49, 1.0]), macrolides (aPR = 0.58, [0.40, 0.86]), fluoroquinolones (aPR = 0.63, [0.37, 1.1]), and chloramphenicols (aPR = 0.69, [0.51, 0.95]).

**Table 3:** Detection of individual ARGs at baseline and the 12-month follow-up in flies.

	Baseline	12-month		Baseline	12-month
Aminoglycoside			Tetracycline		
*armA*			*tetA*		
Control	0% (0/56)	0% (0/53)	Control	71% (40/56)	85% (45/53)
Intervention	0% (0/34)	0% (0/33)	Intervention	88% (30/34)	76% (25/33)
**Chloramphenicol**			*tetB*		
*catA1*			Control	59% (33/56)	62% (33/53)
Control	38% (21/56)	38% (20/53)	Intervention	71% (24/34)	39% (13/33)
Intervention	50% (17/34)	24% (8/33)	**Trimethoprim / Sulfamethoxazole**		
*catB3*			*dfrA17*		
Control	27% (15/56)	42% (22/53)	Control	45% (25/56)	55% (29/53)
Intervention	68% (23/34)	24% (8/33)	Intervention	71% (24/34)	36% (12/33)
*cmlA*			*sul1*		
Control	70% (39/56)	64% (34/53)	Control	100% (56/56)	96% (51/53)
Intervention	71% (24/34)	76% (25/33)	Intervention	100% (34/34)	97% (32/33)
*floR*			*sul2*		
Control	84% (47/56)	83% (47/53)	Control	100% (56/56)	98% (52/53)
Intervention	91% (31/34)	73% (24/33)	Intervention	100% (34/34)	100% (33/33)
**Colistin**			**Β-lactam**		
*mcr-1*			*CTX-M1*		
Control	0% (0/56)	0% (0/53)	Control	7.1% (4/56)	19% (10/53)
Intervention	0% (0/34)	0% (0/33)	Intervention	27% (9/34)	6.1% (2/33)
**Fluoroquinolone**			*CTX-M2-M74*		
*aac6lb_104R*			Control	16% (9/56)	19% (10/53)
Control	11% (6/56)	9.4% (5/53)	Intervention	32% (11/34)	24% (8/33)
Intervention	12% (4/34)	6.1% (2/33)	*CTX-M8-M25*		
*aac6lb_104W*			Control	18% (10/56)	11% (6/53)
Control	55% (31/56)	76% (40/53)	Intervention	24% (8/34)	18% (6/33)
Intervention	79% (27/34)	58% (19/33)	*CTX-M9*		
*gyrA83L*			Control	11% (6/56)	13% (7/53)
Control	20% (11/56)	21% (11/53)	Intervention	21% (7/34)	15% (5/33)
Intervention	8.8% (3/34)	6.1% (2/33)	*NDM*		
*parC80I*			Control	0% (0/56)	0% (0/53)
Control	0% (0/56)	3.8% (2/53)	Intervention	5.8% (2/34)	0% (0/33)
Intervention	2.9% (1/34)	0% (0/33)	*OXA-1*		
*qnrA*			Control	32% (18/56)	43% (23/53)
Control	14% (8/56)	15% (8/53)	Intervention	44% (15/34)	33% (11/33)
Intervention	8.8% (3/34)	18% (6/33)	*OXA-9*		
*qnrB1*			Control	0% (0/56)	7.5% (4/53)
Control	38% (21/56)	45% (24/53)	Intervention	5.8% (2/34)	6.1% (2/33)
Intervention	59% (20/34)	33% (11/33)	*SHV*		
**Macrolide**			Control	61% (34/56)	34% (18/53)
*ermB*			Intervention	56% (19/34)	36% (12/33)
Control	80% (45/56)	77% (41/53)	*VIM*		
Intervention	91% (31/34)	55% (18/33)	Control	0% (0/56)	0% (0/53)
*mphA*			Intervention	0% (0/34)	0% (0/33)
Control	43% (24/56)	55% (29/53)			
Intervention	71% (24/34)	39% (13/33)			

## Discussion

We found evidence that improved onsite sanitation reduced fly counts at latrine entrances, enteric bacteria gene concentrations in flies and the number of ARGs in filth flies from the living environment, though the mean number of ARGs detected per fly remained high 12-months following the delivery of the intervention. This same onsite sanitation intervention did not reduce diarrhea among children nor a range of specific enteric infections [21] so it is unlikely the reduction in ARGs was due to a decrease in antibiotic usage among compound members, though we don’t have data on antibiotic usage to confirm this. The first comprehensive assessment of the global burden of AMR found the highest burden in low-income settings and argued that sanitation infrastructure is likely fundamental to combat the spread of AMR [1]. Our results offer promising empirical evidence that improving onsite sanitation in informal settlements may reduce AMR carried by flies, which is mediated by reductions in enteric bacteria [1]. However, we were unable to directly connect our results with human health outcomes, nor did we observe any difference in fly counts in food preparations areas, which is more exposure relevant than latrine entrances.

The protective effect we observed may be because the intervention served as a physical barrier between flies and human waste. The sanitation intervention included a pour flush toilet and ventilation pipe covered by mesh netting. Physical barriers at intervention compounds may have prevented flies from feeding on fecal wastes, which contain enteric bacteria that may possess ARGs. While we lack observational data on water seals and ventilation pipe screens during the 12-month follow-up, we infrequently observed ventilation pipe covers (n = 13/48) among a small subset of intervention compounds two to three years following delivery of the intervention [26]. As pipe covers were part of the intervention this observation indicates that degradation occurred. Degradation of the intervention’s physical barriers would have reduced the intervention’s ability to separate flies from fecal waste and possibly the efficacy of the intervention. In addition, these species of flies are highly mobile and have a potential range of several kilometers in a single day [7, 8]. The sanitation intervention was delivered to clusters of households and not community wide, meaning that intervention and control compounds were present in the same neighborhoods and sometimes were even adjacent. Given the transience of flies it is even more striking that we observed a protective effect, and it is possible that community wide interventions may have a larger impact than we observed.

Flies are effective mechanical vectors that may transport microbes on their outer body and in their alimentary canal, where some bacteria can multiply [51, 52]. Their capacity to quickly disperse large quantities of these organisms–including pathogens–throughout the living environment demonstrates a critical need for fly control. Controlled feeding studies have demonstrated flies are capable of transferring >10^5^ CFU *E*. *coli* to common food items in a 30-minute period [53]. In rural Bangladesh Ercumen *et al*. 2017 found a 1-log_10_ increase in *E*. *coli* carried by flies caught in the living environment was associated with a 0.21 log_10_ increase in *E*. *coli* concentration in child food [54]. The high concentration and prevalence of ARGs we detected, as well as a high prevalence of resistant isolates, offers evidence that flies may act as a mechanical vector of AMR transmission. Improved sanitation may then serve a dual public health purpose by reducing the environmental transmission of enteric pathogens and AMR.

In Capone *et al*. 2023 we evaluated enteric pathogens carried by flies using the same samples as this study [24]. We detected genes specific to enteropathogenic *E*. *coli* (21%, 37/176), enteroaggregative *E*. *coli* (19%, 33/176), enterotoxigenic *E*. *coli* (15%, 27/176), *Shigella* / enteroinvasive *E*. *coli* (4.0%, 7/176), *Vibrio cholerae* (2.8%, 5/176), shiga-toxin producing *E*. *coli* (1.7%, 3/176), and *Campylobacter jejuni*/*coli* (1.1%, 2/176) in flies, but did not detect *Salmonella* spp. or *Clostridium difficile*. The prevalence of ≥1 bacterial pathogen gene remained constant in the control group from baseline (39%, 22/56) to the 12-month follow-up (40%, 21/53), but decreased in the intervention group from 47% (16/34) at baseline to 24% (8/33) 12-months later. While the intervention was associated with a 42% reduction (aPR = 0.58, [0.25, 1.3]) in the detection of ≥1 bacterial pathogen gene in flies, the confidence interval was wide, which indicates the intervention may have had no effect or even increased the prevalence of ≥1 bacterial pathogen gene in flies.

Previous assessments of community wide fly control measures found similar reductions in fly counts and observed improvements in health outcomes. A crossover study of residual dichlorodiphenyltrichloroethane (DDT) application to public areas (e.g., fields, barns, and latrines) in rural Texas during the 1940s observed a 90% reduction in fly counts and observed a reduction in *Shigella* infections among community members [11]. Though even in the 1940s insect resistant to insecticides was observed [55], as well as a growing recognition of DDT’s deleterious environmental and health effects [56, 57]. Studies conducted in the 1990s in rural Pakistan and the Gambia found that community-scale insecticide application reduced childhood diarrhea by 23% and 24%, respectively [58, 59]. A study of US military bases found intensive fly control via baited traps reduced clinic visits attributable to diarrhea by 42% and *Shigella* seroconversion by 76% [60]. A 2016 systematic review of onsite sanitation interventions–which are typically implemented at the household level–found evidence that onsite sanitation was associated with reductions in fly counts [61]. Our study complements these previous assessments by providing suggestive evidence that onsite sanitation may reduce enteric bacteria and antimicrobial resistance genes carried by flies.

Widespread use of antibiotics in Maputo may be driving AMR emergence and spread. Antibiotics can be purchased without a prescription from pharmacies and in informal markets [62]. Among those who purchased antibiotics without a prescription in Maputo, Mate *et al*. 2019 reported that only 10% completed the full course [62]. Multiple context specific factors drive individuals to self-medicate with antibiotics, including the poor quality of care at health care facilities and long wait times, advice from pharmacists, and previous positive experiences taking antibiotics [63]. While not systematically collected, Torres *et al*. 2019 reported that Maputo pharmacists frequently dispense amoxicillin, azithromycin, tetracycline, and trimethoprim/sulfamethoxazole [64]. This usage aligns with our genotypic and phenotypic results; we observed the highest prevalence of resistant *Enterobacteriaceae* isolates for trimethoprim/sulfamethoxazole and the corresponding ARGs (i.e., sul1 and sul2) were detected in nearly all flies. Reducing the spread of AMR in Mozambique–and similar contexts–will require multi-faceted interventions. Limiting the use of antibiotic when not necessary for human and animal health is fundamental. Other key interventions will also be necessary, including hospital-based control programs, preventing infections through vaccination, and functional sanitation infrastructure [1].

Our study had several limitations. First, we only assessed phenotypic resistance at the pre-intervention time point, which prevented us from evaluating if the intervention reduced phenotypic resistance of isolates in addition to genotypic resistance. Further, we cultured isolates from frozen flies, which may have resulted in die-off that could have been differential between species. Second, we tested multiple isolates per pair of flies for phenotypic resistance, but only differentiated them based on morphology and without confirmation that these isolates were different strains. It is possible some of the isolates we picked from pairs of flies were the same species. In addition, some bacterial species are also intrinsically resistant to some antibiotics, which limited our scope to describing the phenotypic resistance profile of the isolates assessed. Third, we did not include an antibiotic in the TSB media used to grow isolates, which may have resulted in the loss of plasmid mediated resistance and caused us to underestimate phenotypic resistance. Fourth, the intervention was not randomly allocated, but we accounted for this via the controlled before-and-after study design [21]. Fifth, our methods were limited to pre-specified PCR assays and did not assess the entire resistome. Sequencing methods may have identified other ARGs, such as genes encoding resistance to colistin which we did not detect via PCR but were observed in our phenotypic assays. In addition, we sterilized tweezers between compounds and not individual flies, which may have led to contamination between flies from the same compound. Sixth, due to manufacturer discontinuation, we were forced to change fly traps between the baseline and 12-month assessment. Different sticky fly traps may work better or worse at catching flies and may be better suited at catching certain species of flies. However, our approach was applied equally across study arms, which reduces the bias that may have resulted from using different traps. Seventh, we did not identify flies beyond visual classification as a house fly or green bottle fly. Fly species often vary in their preferred feeding material and fly control recommendations may depend on the specific species of interest [7, 8, 65]. Finally, we may have underestimated the impact on AMR in flies because we estimated the reduction in ARGs per individual fly, but we also observed a large reduction in fly counts at latrine entrances and fly counts were not considered in the ARG regression model.

## Conclusions

AMR is a threat to global public health whose scope and increasing burden merits better monitoring and evaluation to assess trends and develop strategies to reduce its spread [66]. We observed a high level of genotypic resistance among flies and phenotypic resistance among *Enterobacteriaceae* isolates, but also found that a shared onsite sanitation intervention (that included basic fly control) was associated with a reduction in the ARGs carried by flies compared to a control group not receiving sanitation upgrades. Fly control strategies are well established, including physical barriers (e.g., pour flush systems, drop hole covers, and ventilation pipe covers) and insecticide spraying [58]. These results–alongside previous work on flies in other low- and middle-income countries [15, 67]–offers evidence that the contribution of flies to the transmission of sanitation-related microbes and AMR may be under-recognized. The design of sanitation infrastructure and accompanying services should include sustained and effective fly control measures.

## Supporting information

S1 Text Extraction protocol. (PDF)

S1 Table TAC performance. (PDF)

S2 Table MIQE checklist. (PDF)

S3 Table Phenotypic assessment. (PDF)

S4 Table Zones of inhibition. (PDF)

S5 Table Fly count by month. (PDF)

S6 Table Fly count by location. (PDF)

S1 Fig qPCR plots. (PDF)

S2 Fig Quantitative results. (PDF)

S3 Fig Multidrug resistance of isolates. (PDF)
