# Supplementary material for: Sanitation in urban areas may limit the spread of antimicrobial resistance via flies
Source: PLoS One. 2024 Mar 20;19(3):e0298578. doi: 10.1371/journal.pone.0298578 (PMC10954131; doi:10.1371/journal.pone.0298578)
Supplement: S1 Text — (PDF) [file pone.0298578.s001.pdf]

S1 Text. Extraction protocol

Nucleic acids were extracted from one fly corresponding to each compound where we caught ≥1 fly and had flies available in Atlanta to do so (i.e., some flies remained in Maputo). Flies were first visually inspected and categorized as a house fly, bottle fly, or other based on illustrative diagrams in Greenberg 1973 [1]. For compounds with ≥1 fly available for extraction, we developed a heuristic for fly selection. We systematically prioritized the extraction of house flies over green bottle flies, then flies from food preparation areas over latrine entrances due to the near-universal prevalence of houseflies on traps, and because the food preparation area was more exposure-relevant than the latrine entrance (see figure below). If multiple flies met our criteria (e.g., multiple houseflies caught at a compound's food preparation area) we then randomly selected a single fly for extraction from this pool of eligible flies.

We used the Qiagen DNeasy Blood and Tissue Kit (Qiagen, Hilden, Germany) to extract total nucleic acids from 188 individual flies. First, we bead beat flies for four cycles of 45 seconds in bead beating tubes containing 67 mg of each of three sizes of glass beads (2 mm, 425-600μm, 150-212μm, Sigma, St. Louis, MO) and 180 μL Qiagen Buffer ATL using a Mini-BeadBeater-16 (BioSpec, Bartlesville, OK). After addition of 180 Buffer μL ATL, 40 μL proteinase K (Qiagen, Hilden, Germany), and 6 μL carrier RNA (Qiagen, Hilden, Germany) to each tube we incubated the flies for three hours at 56°C. This pre-treatment step was adapted from the recommended protocol for animal tissue and has been used widely for extractions from ticks [2–5]. Then, we proceeded with extraction following the manufacturer's protocol. We spiked in approximately 10^7 gene copies of bacteriophage MS2 (ATCC, Manassas, VA), an RNA phage, and 10^6 copies of a DNA gBlock (IDT, Coralville, IA) prior to bead-beating as our extraction positive controls respectively [6]. On each day of extractions, we included at least one negative extraction control (i.e., nuclease free water).

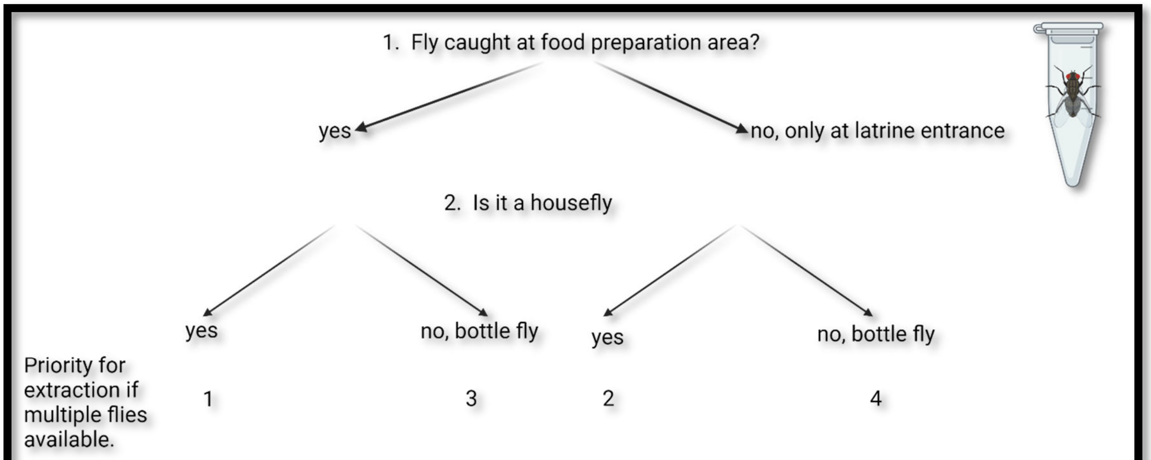

Fly selection priorities (in order of priority)

1. Houseflies prioritized over bottle flies
2. Flies caught at food preparation areas over those caught at the latrine entrance
3. If multiple flies met a single priority level (e.g., two houseflies were caught at the food preparation area), then one was randomly selected
