# Supplementary material for: Sanitation in urban areas may limit the spread of antimicrobial resistance via flies
Source: PLoS One. 2024 Mar 20;19(3):e0298578. doi: 10.1371/journal.pone.0298578 (PMC10954131; doi:10.1371/journal.pone.0298578)
Supplement: S1 Table — (PDF) [file pone.0298578.s002.pdf]

S1 Table. TAC Performance

Target	Class	Gene	y-intercept	R^2	Efficiency	LOD (gc/µL)*	Ref
CTX-M1	AMR- beta-lactam	CTX-M1	36.9	1.000	96%	2.4	[7]
CTX-M2-M74	AMR- beta-lactam	CTX-M2-M74	42.4	0.985	108%	24	[7]
CTX-M8-M25	AMR- beta-lactam	CTX-M25	36.3	1.000	96%	24	[7]
CTX-M9	AMR- beta-lactam	CTX-M9	40.6	0.984	115%	24	[7]
NDM	AMR- beta-lactam	NDM	40.9	0.998	98%	24	[7]
OXA-1	AMR- beta-lactam	OXA-1	40.7	0.999	97%	24	[7]
OXA-9	AMR- beta-lactam	OXA-9	40.7	1.000	95%	24	[7]
SHV	AMR- beta-lactam	SHV	36.0	0.999	97%	24	[7]
VIM	AMR- beta-lactam	VIM	39.7	0.999	97%	24	[7]
aac6lb_104R	AMR- fluoroquinolone	aac(6')-lb-104R	39.6	0.999	90%	24	[7]
aac6lb_104W	AMR- fluoroquinolone	aac(6')-lb-104W	40	0.998	91%	24	[7]
armA	AMR- aminoglycoside	armA	39.1	0.998	89%	24	[7]
catA1	AMR- chloramphenicol	catA1	40.8	0.996	92%	24	[7]
catB3	AMR- chloramphenicol	catB3	40.2	0.996	92%	24	[7]
cmlA	AMR- chloramphenicol	cmlA	39.6	0.981	97%	24	[7]
dfrA17	AMR- trimethoprim/sulfa	dfrA17	40.5	0.999	95%	24	[7]
ermB	AMR- macrolide	ermB	40.8	0.999	99%	2.4	[7]
floR	AMR- chloramphenicol	floR	39.9	0.980	98%	24	[7]
gyrA83L	AMR- fluoroquinolone (ciprofloxacin)	gyrA83L-Esh	42	0.999	92%	2.4	[7]
gyrA83S	AMR- fluoroquinolone (ciprofloxacin)	gyrA83S-Esh	39.6	1.000	92%	2.4	[7]
intl1	Mobile genetic element	intl1	39.8	0.998	94%	24	[7]
mcr-1	AMR- colistin	mcr-1	41.3	0.977	86%	2.4	[7]
mphA	AMR- macrolide	mphA	40.2	0.999	99%	24	[7]

parC80I	AMR- fluoroquinolone (ciprofloxacin)	parC80I-Esh	38.8	0.999	95%	24	[7]
parC80S	AMR- fluoroquinolone (ciprofloxacin)	parC80S-Esh	39.7	0.995	91%	24	[7]
qnrA	AMR- fluoroquinolone	qnrA	39.1	0.998	92%	24	[7]
qnrB1	AMR- fluoroquinolone	qnrB1	40.4	0.999	96%	24	[7]
sul1	AMR- trimethoprim/sulfa	sul1	37.6	0.996	94%	24	[7]
sul2	AMR- trimethoprim/sulfa	sul2	39.9	0.998	94%	24	[7]
tetA	AMR- tetracycline	tetA	39.4	0.999	95%	2.4	[7]
tetB	AMR- tetracycline	tetB	41.2	1.000	95%	2.4	[7]
enteric 16S	NA	16S	39.8	0.960	89%	2.4	[8]

*LOD = Limit of Detection; We used droplet digital PCR to quantify our combined positive control. Then we ran a dilution series to calculate the limit of detection, which is reported as the lowest concentration with a positive detection. Performance of bacterial pathogen assays is reported in Capone et al. 2022[9]
