# Supplementary material for: Sanitation in urban areas may limit the spread of antimicrobial resistance via flies
Source: PLoS One. 2024 Mar 20;19(3):e0298578. doi: 10.1371/journal.pone.0298578 (PMC10954131; doi:10.1371/journal.pone.0298578)
Supplement: S2 Table — (PDF) [file pone.0298578.s003.pdf]

S2 Table. MIQE Checklist

Group	Item	Response
Experimental Design	Definition of experimental and control groups	The intervention group received the onsite sanitation intervention, and the control group did not.
	Number within each group	Baseline: Control (n=56), Intervention (n=34)<br>12-month: Control (n=53), Intervention (n=33)
	Assay carried out by the core or investigator's laboratory?	Assays were carried out by the Brown Lab at UNC-Chapel Hill.
	Acknowledgment of authors' contributions	DC: Conceptualization, Formal analysis, Investigation, Writing - Original Draft<br>OC: Methodology, Funding acquisition, Writing - Review & Editing<br>AF: Formal analysis, Writing - Review & Editing<br>VI: Investigation, Writing - Review & Editing<br>SI: Methodology, Writing - Review & Editing<br>IK: Writing - Review & Editing<br>JK: Methodology, Investigation, Writing - Review & Editing<br>RN: Supervision, Resources, Writing - Review & Editing<br>JB: Conceptualization, Funding acquisition, Writing - Review & Editing
Sample	Description	Samples were houseflies and green bottle flies caught at latrine entrances and food preparation areas

		among low-income informal settlements in Maputo, Mozambique.
	Volume/mass of sample processed	Fly Mass Summary (mg):<br>Mean = 9.8<br>Median = 8.90 (IQR= 4.80, 13)<br>Min = 0.40<br>Max = 52.90
	Processing procedure	Methods described in the main text.
	If frozen, how and how quickly?	Samples were frozen at -80C within six hours of collection.
	If fixed, with what and how quickly?	Not fixed.
	Sample storage conditions and duration	Samples remained at -80C approximately five years before analysis.
Nucleic acid extraction	Procedure and/or instrumentation	Methods described in the main text.
	Name of kit and details of any modifications	Methods described in the main text.
	Source of additional reagents used	Methods described in the main text.
	Details of DNase or RNase treatment	Not treated
	Contamination assessment (DNA or RNA)	One extraction negative control was included per day of extractions.
	Nucleic acid quantification	Nucleic acid was not quantified since most of the nucleic acid would have been from the fly itself.
	Instrument and method	Manual extractions were performed in a biological safety cabinet.
	Inhibition testing (Cq dilutions, spike, or other)	Inhibition was monitored using spike in control: RNA bacteriophage

		MS2 (ATCC, Manassas, VA) and a synthetic DNA sequence (IDT, Coralville, IA)
Reverse transcription	Complete reaction conditions	One-step reverse transcription
	Amount of RNA and reaction volume	Reaction Volume = 1.5 µL
	Reverse transcriptase and concentration	ArrayScript™ Reverse Transcriptase
	Temperature and time	45°C for 20 minutes
	Manufacturer of reagents and catalogue numbers	Applied Biosystems, AgPath-ID™ One-Step RT-PCR Reagents Catalog number: 4387391
qPCR target information	Gene symbol	Provided in Table S1
	In silico specificity screen (BLAST, and so on)	We BLASTed all assays to confirm specificity before ordering the custom TAC.
qPCR oligonucleotides	Primer sequences	Primer sequences were taken from Pholwat et al. 2019[7]
	Probe sequences	Primer sequences were taken from Pholwat et al. 2019[7]
	Location and identity of any modifications	No modifications
	Manufacturer of oligonucleotides	ThermoFisher Scientific
qPCR protocol	Complete reaction conditions	45°C for 20 min and 95°C for 10 min, followed by 45 cycles of 95°C for 15 s and 60°C for 1 min
	Reaction volume and amount of cDNA/DNA	100µL reactions were prepared, containing 60uL of mastermix and 40µL of template. This corresponds to 0.6µL of template and 0.9µL of mastermix per reaction well.
	Primer, (probe), Mg2+, and dNTP concentrations	All assays contained the same concentrations of primers (900

		nanomolar) and probe (250 nanomolar). The Mg2+ and dNTP concentrations are not listed in the in the User Guide.
	Polymerase identity and concentration	AmpliTaq Gold™ polymerase
	Buffer/kit identity and manufacturer	AgPath-ID™ One-Step RT-PCR Reagents
	Additives (SYBR Green I, DMSO, and so forth)	No additives
	Manufacturer of plates/tubes and catalog number	ThermoFisher Scientific
	Complete thermocycling parameters	45°C for 20 min and 95°C for 10 min, followed by 45 cycles of 95°C for 15 s and 60°C for 1 min
	Reaction setup (manual/robotic)	Manual set-up in a disinfected dead air box (10% bleach with fifteen minutes of contact time, UV for fifteen minutes, and a final cleaning step with 70% ethanol)
	Manufacturer of qPCR instrument	ThermoFisher Scientfic
qPCR validation	Evidence of optimization (from gradients)	See Pholwat et al. 2019[7]
	Specificity (gel, sequence, melt, or digest)	See Pholwat et al. 2019[7]
	Calibration curves with slope and y intercept	See Table S1
	PCR efficiency calculated from slope	See Table S1
	r2 of calibration curve	See Table S1
	Evidence for LOD	See Table S1
Data analysis	qPCR analysis program (source, version)	QuantStudio Real-Time PCR Software V1.2 CDC

	Method of Cq determination	Manual thresholding
	Results for NTCs	We observed no amplification before at Ct of 40 in our two PCR negative controls.<br><br>Among the 12 negative extraction controls, we observed no amplification before a Ct of 40.
	Description of normalization method	Normalized per individual fly
	Statistical methods for results significance	Methods described in the main text
	Software (source, version)	R Studio V2.2.2
