# Supplementary material for: Sanitation in urban areas may limit the spread of antimicrobial resistance via flies
Source: PLoS One. 2024 Mar 20;19(3):e0298578. doi: 10.1371/journal.pone.0298578 (PMC10954131; doi:10.1371/journal.pone.0298578)

S3 Table. Phenotypic Assessment of Enterobacterales isolates

**Organisms**	**Growth**
Enterobacter aerogenes	Mucoid, pink to pinkish-red
Escherichia coli	Pinkish red with bile precipitate
Salmonella Enteritidis	Colorless to orangish-yellow

Note: Grown on violet red bile agar[10]. This a figure is similar but not identical to the original image and is therefore for illustrative purposes only.

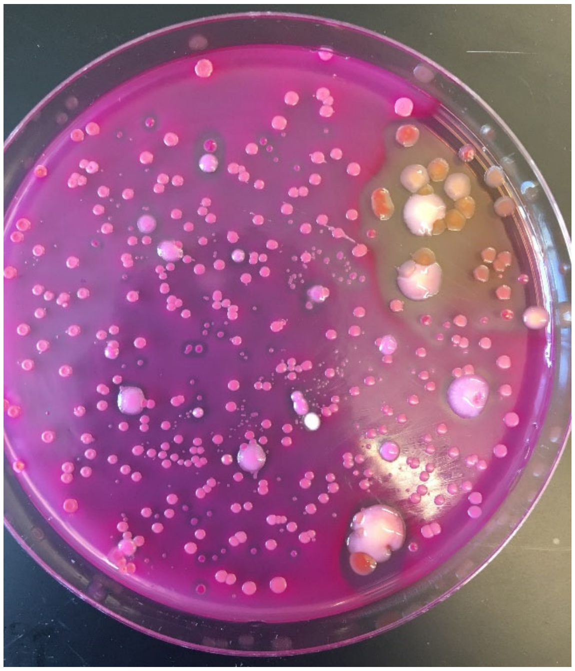

Supplement: S3 Table — (PDF) [file pone.0298578.s004.pdf]
