# Supplementary material for: Sanitation in urban areas may limit the spread of antimicrobial resistance via flies
Source: PLoS One. 2024 Mar 20;19(3):e0298578. doi: 10.1371/journal.pone.0298578 (PMC10954131; doi:10.1371/journal.pone.0298578)
Supplement: S4 Table — (PDF) [file pone.0298578.s005.pdf]

S4 Table. Zones of inhibition

Antibiotics	Antibiotic Disk Content	Susceptible (mm)	Intermediate (mm)	Resistant (mm)
Ciprofloxacin **(CIP)**	5 µg	≥21	16-20	≤15
Streptomycin **(S)**	10 µg	≥15	12-14	≤11
Levofloxacin **(LEV)**	5 µg	≥17	14-16	≤13
Chloramphenicol **(C)**	30 µg	≥18	13-17	≤12
Colistin **(CT)**	10 µg	≥11	8-11	≤8
Azithromycin **(AZM)**	15 µg	≥13	12-13	≤12
Tetracycline **(TE)**	30 µg	≥15	12-14	≤11
Trimethoprim-Sulfamethoxazole **(SXT)**	1.25/ 23.75 µg	≥16	11-15	≤10
Ampicillin-Sulbactam **(SAM)**	10/10 µg	≥15	12-14	≤11
Ceftazidime –Avibactam **(CAZ/AV1)**	30/20 µg	≥21	20-21	≤ 20

Note: Categories for zones of inhibition were taken from CLSI guidelines[11,12]. This a figure is similar but not identical to the original image and is therefore for illustrative purposes only.
