# Supplementary material for: Sanitation in urban areas may limit the spread of antimicrobial resistance via flies
Source: PLoS One. 2024 Mar 20;19(3):e0298578. doi: 10.1371/journal.pone.0298578 (PMC10954131; doi:10.1371/journal.pone.0298578)
Supplement: S5 Table — (PDF) [file pone.0298578.s006.pdf]

S5 Table. Fly Count by Month

				Fly Count (log10)		Fly Count (continuous)	
Phase	Year	Month	Compounds Visited	Average	SD	Average	SD
Baseline	2015	April	4	0.74	0.74	7.8	5.0
	2015	May	7	0.25	0.25	11.9	17.8
	2015	June	28	0.82	0.82	18.2	22.2
	2015	July	43	0.38	0.38	12.8	19.1
	2015	August	11	0.78	0.78	13.4	16.4
	2015	September	2	1.61	1.61	48.0	35.4
	2015	October	9	1.21	1.21	23.1	19.2
	2016	January	33	1.00	1.00	14.4	13.0
12-Month	2016	April	4	0.69	0.69	18.5	22.6
	2016	June	12	-0.24	-0.24	3.8	7.2
	2016	July	45	0.23	0.23	5.0	6.0
	2016	August	14	0.18	0.18	4.0	4.4
	2016	September	8	0.46	0.46	4.8	3.8
	2016	October	3	0.62	0.62	5.0	3.6
	2016	November	1	0.30	NA	2.0	NA
	2017	January	5	0.05	0.05	3.8	4.3
	2017	February	21	-0.62	-0.62	1.0	2.1
	2017	March	6	-1	NA	0	NA

Note: We imputed non-detects (i.e., no flies were caught) to 0.1 to $\log_{10}$ transform the data. NA implies there were insufficient flies or compounds visited to calculate a standard deviation
