# Supplementary material for: Sanitation in urban areas may limit the spread of antimicrobial resistance via flies
Source: PLoS One. 2024 Mar 20;19(3):e0298578. doi: 10.1371/journal.pone.0298578 (PMC10954131; doi:10.1371/journal.pone.0298578)
Supplement: S6 Table — (PDF) [file pone.0298578.s007.pdf]

S6 Table. Fly Count by Location

Phase	Arm	Location	Mean Number of Flies Caught
Baseline	Control	Latrine	3.7
	Intervention	Latrine	5.8
	Control	Food Preparation	9.7
	Intervention	Food Preparation	13
12-month follow-up	Control	Latrine	2.5
	Intervention	Latrine	0.8
	Control	Food Preparation	2.1
	Intervention	Food Preparation	2.4
			Prevalence of ≥1 fly caught
Baseline	Control	Latrine	46%
	Intervention	Latrine	54%
	Control	Food Preparation	76%
	Intervention	Food Preparation	85%
12-month follow-up	Control	Latrine	49%
	Intervention	Latrine	20%
	Control	Food Preparation	31%
	Intervention	Food Preparation	44%
