# Supplementary figures and images for: Sanitation in urban areas may limit the spread of antimicrobial resistance via flies
Source: PLoS One. 2024 Mar 20;19(3):e0298578. doi: 10.1371/journal.pone.0298578 (PMC10954131; doi:10.1371/journal.pone.0298578)

S1 Fig. qPCR plots

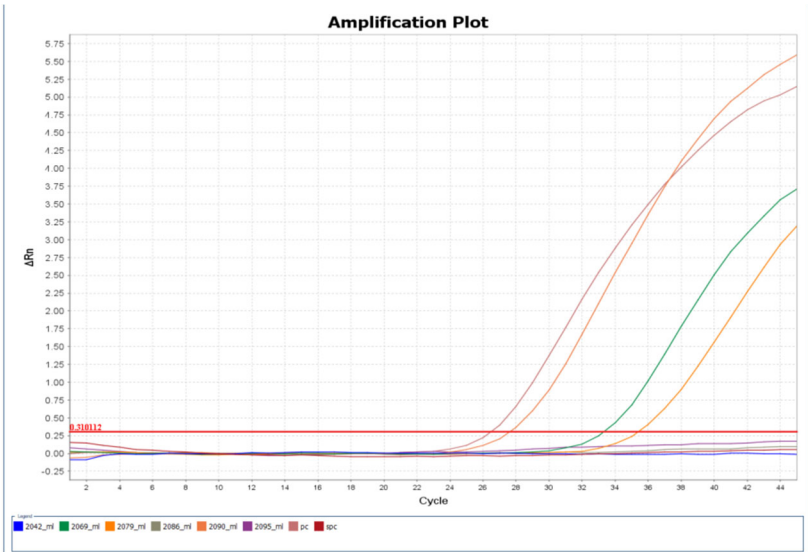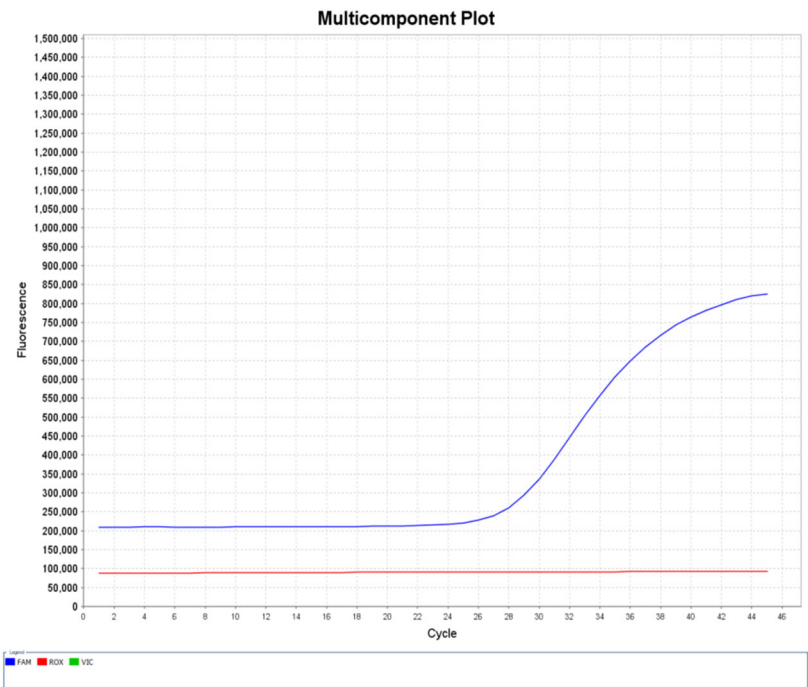

Supplement: S1 Fig — (PDF) [file pone.0298578.s008.pdf]

S2 Fig. Quantitative results

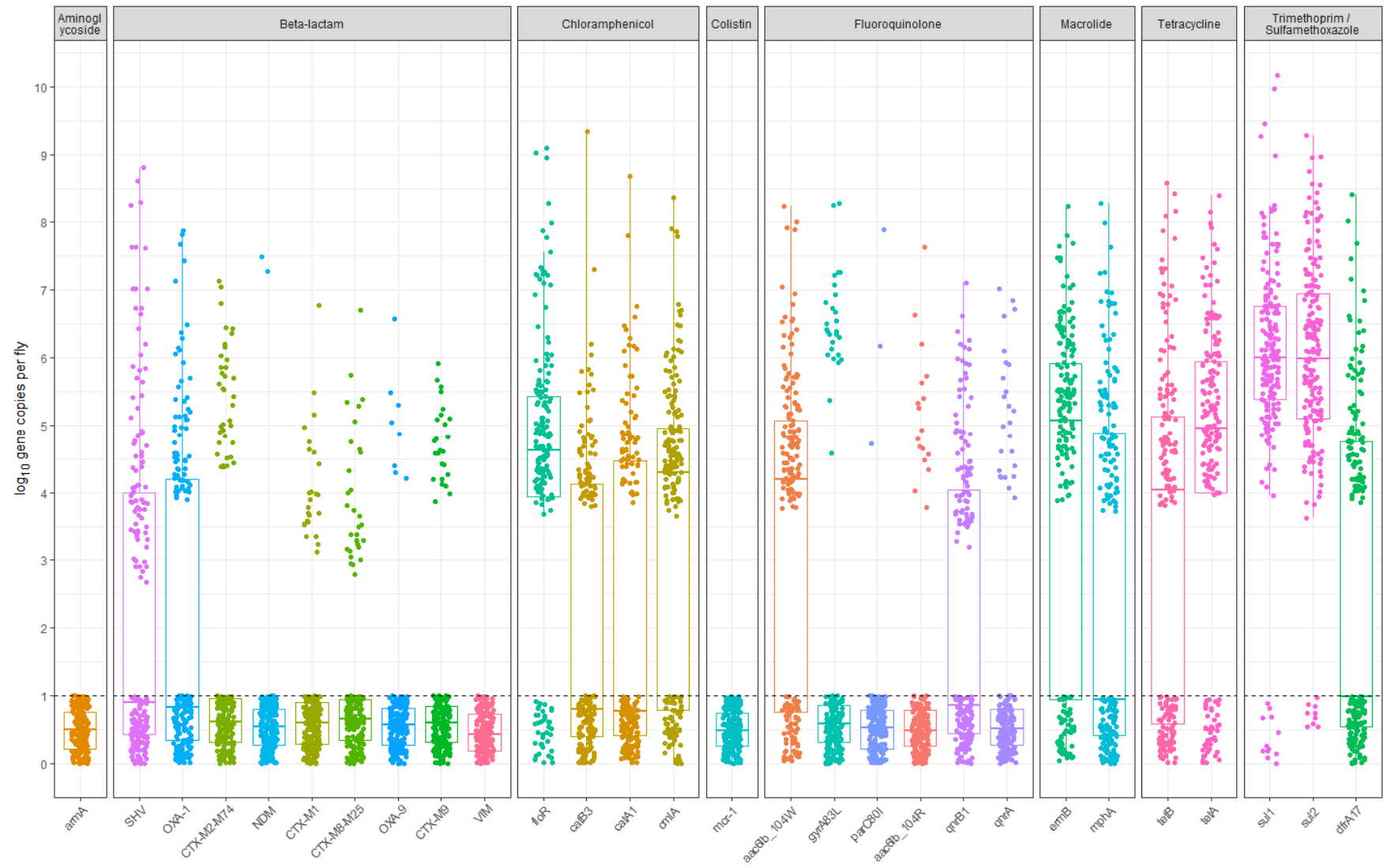

Note: Non-detects were imputed and are shown below the dotted line

Supplement: S2 Fig — (PDF) [file pone.0298578.s009.pdf]
