# Supplementary material for: Sanitation in urban areas may limit the spread of antimicrobial resistance via flies
Source: PLoS One. 2024 Mar 20;19(3):e0298578. doi: 10.1371/journal.pone.0298578 (PMC10954131; doi:10.1371/journal.pone.0298578)
Supplement: S3 Fig — (PDF) [file pone.0298578.s010.pdf]

S3 Fig. Multiantibiotic resistance of isolates

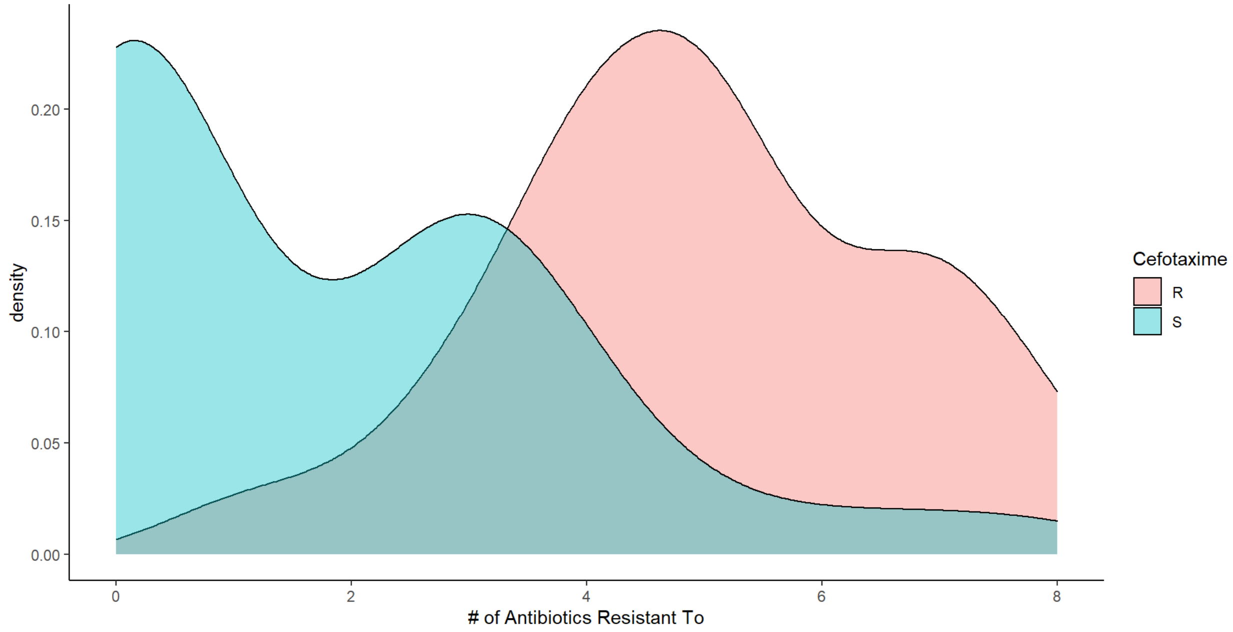

Note: R = resistant. S = susceptible. The x-axis in the figure excludes cefotaxime
